# A comprehensive genomic catalog from global cold seeps

**DOI:** 10.1038/s41597-023-02521-4

**Published:** 2023-09-08

**Authors:** Yingchun Han, Chuwen Zhang, Zhuoming Zhao, Yongyi Peng, Jing Liao, Qiuyun Jiang, Qing Liu, Zongze Shao, Xiyang Dong

**Affiliations:** 1 Key Laboratory of Marine Genetic Resources, Third Institute of Oceanography, Ministry of Natural Resources, Xiamen, 361005 China; 2 School of Marine Sciences, Sun Yat-Sen University, Zhuhai, 519082 China; 3 Southern Marine Science and Engineering Guangdong Laboratory (Zhuhai), Zhuhai, 519000 China

**Keywords:** Environmental microbiology, Metagenomics

## Abstract

Cold seeps harbor abundant and diverse microbes with tremendous potential for biological applications and that have a significant influence on biogeochemical cycles. Although recent metagenomic studies have expanded our understanding of the community and function of seep microorganisms, knowledge of the diversity and genetic repertoire of global seep microbes is lacking. Here, we collected a compilation of 165 metagenomic datasets from 16 cold seep sites across the globe to construct a comprehensive gene and genome catalog. The non-redundant gene catalog comprised 147 million genes, and 36% of them could not be assigned to a function with the currently available databases. A total of 3,164 species-level representative metagenome-assembled genomes (MAGs) were obtained, most of which (94%) belonged to novel species. Of them, 81 ANME species were identified that cover all subclades except ANME-2d, and 23 syntrophic SRB species spanned the Seep-SRB1a, Seep-SRB1g, and Seep-SRB2 clades. The non-redundant gene and MAG catalog is a valuable resource that will aid in deepening our understanding of the functions of cold seep microbiomes.

## Background & Summary

Cold seeps occur on continental margins worldwide. At these sites, methane-rich fluids migrate from the deep subsurface to the sediment-water interface^1^. Methane is a climate-active greenhouse gas that is approximately 30 times more potent than carbon dioxide^2^. In seep sediments, methane can be consumed through the process of anaerobic oxidation of methane (AOM). This process removes approximately 90% of the methane produced globally in marine sediments, acting as an efficient methane filter^3,4^. As a consequence, these seeps are critical in regulating the amount of methane released into the overlying waters and atmosphere, and they play a vital role in mitigating global warming. AOM is performed by anaerobic methanotrophic archaea (ANME). Normally, ANME rely on a syntrophic partner to couple CH_4_ oxidation to the reduction of terminal electron acceptors, such as sulfate, iron, nitrate, and manganese^5,6^. AOM coupled to sulfate reduction is the primary biological process in seep sediments since sulfate is the dominant anion present at the marine sediment-water interface. High rates of AOM fueled by near-saturated methane concentrations would rapidly consume sediment pools of any individual electron acceptor, creating unique geobiological engines that contribute significantly to local and global biogeochemical cycles^1^.

Cold seeps are deep-sea oases that support immense biodiversity and where specialization and adaptation create extraordinary lifestyles^1^. However, the majority of microorganisms found in seeps have not yet been characterized^7^. Culture-independent metagenomic techniques are the key to unraveling the genetic diversity and metabolic potential of uncharacterized microbes and have been applied to identify thousands of microorganisms and their metabolic versatility. Recently, the microbial community and function of cold seep sediments have been increasingly studied with metagenomes obtained from different sea areas^7–10^. However, there are no large-scale gene and genome catalogs available for the microbiome of global cold seeps. A comprehensive gene and genome catalog of cold seeps could serve as a reference for mining novel genetic resources in the deep sea, including various natural products with diverse bioactivities (e.g., antibiotic dixiamycins and immune-enhancing exopolysaccharides)^11,12^.

ANME and their syntrophic sulfate-reducing bacteria (SRB) partners play a crucial role in the regulation of both the carbon and sulfur cycles of seeps. Through their mutualistic interactions, they perform AOM, leading to a reduction in methane release and the generation of inorganic carbon and sulfide. These processes are of significant importance for both local and global biogeochemical cycles, underscoring the essential role of these microorganisms in deep-sea ecosystems. Although previous findings have revealed various lineages of ANME and SRB in seep sediments^13–15^, there is currently no a comprehensive genome catalog of these lineages in cold seep sediments globally. Extensive, high-quality reference genomes of the global seep microbiome could improve the resolution and accuracy of taxonomic and functional analyses and provide the opportunity for large-scale comparative genomics^16–19^, especially for elucidating the physiological basis of ANME-SRB interactions.

**Fig. 1:**
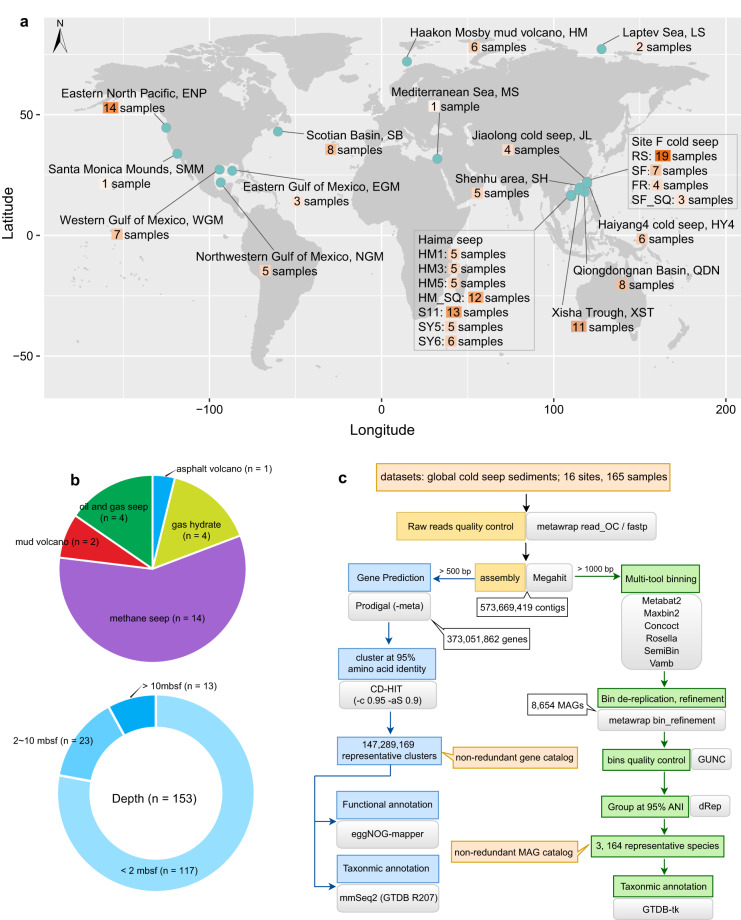
Overview of the studied areas and bioinformatics workflow. (**a**) Geographic distribution of the 16 global cold seep sites where metagenomic sequencing data were collected. The map was drawn using the maptools and ggplot2 packages in R v4.0.3. (**b**) Numbers and proportions of cold seep samples classified according to their types and depths. (**c**) Overview of the computational pipeline used to generate the non-redundant gene and MAG catalogs.

**Fig. 2:**
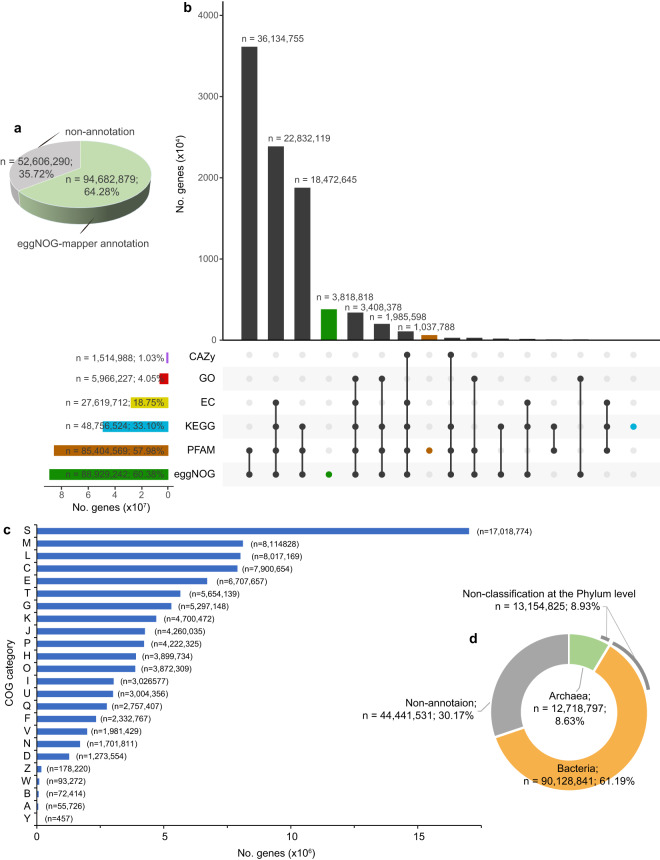
Functional and taxonomic characterization of the non-redundant gene catalog. (**a**) An overview of annotations for the non-redundant gene catalog. Non-annotation indicates that these genes were not annotated in at least one of the following databases: eggNOG, Pfam, KEGG, EC, GO and CAZy. (**b**) Number of genes with functional annotations across the six functional databases. Vertical bars represent the number of genes unique (color) to each functional database or shared (black) between different functional databases. Horizontal bars in the lower panel indicate the total number of genes with functional annotations in each database. (**c**) Functional annotations at the COG category level. S: Function unknown. (**d**) Breakdown of taxonomic classifications for the non-redundant gene catalog.

Here, we collected metagenomic sequence data from 165 sediment samples at 16 cold seeps across the Pacific, Atlantic, and Arctic Oceans (Fig. 1), encompassing gas hydrates (n = 4), methane seeps (n = 14), oil and gas seeps (n = 4), mud volcanoes (n = 2) and asphalt volcanoes (n = 1). The sediment samples span different depths and redox conditions, from the oxic sediment-water interface to anoxic layers down to 68.55 m below the sea floor (mbsf) (Supplementary Table 1). The non-redundant gene catalog was constructed from these metagenomes, comprising a total of 147,289,169 protein clusters (Fig. 2). The mapping ratios of the non-redundant gene catalog to clean reads of the 165 metagenomes averaged 62%. This is the most comprehensive gene catalog generated from the cold seep sediment microbiome to date, corresponding to half the size of the global microbial gene catalog (GMGC v1; 303 million)^16^, the size of the global topsoil microbiome gene catalog (~160 million)^20^, three times the size of the ocean microbial reference gene catalog (OM-RGC v2; ~47 million)^21^, and six times the size of the Tibetan Glacier gene catalog (TG2G; ~25 million)^17^.

**Fig. 3:**
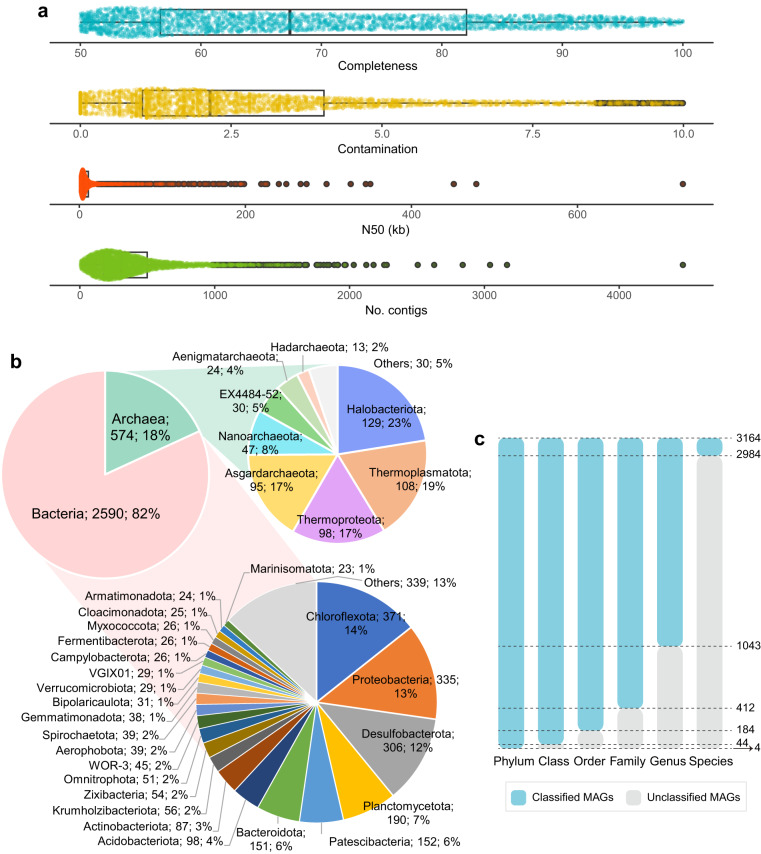
Quality and novelty of non-redundant MAGs. (**a**) Genome statistics for the representative species of non-redundant MAGs. (**b**) Taxonomic classification (domain and phylum levels) of the species-level representative MAGs. (**c**) Taxonomic novelty of the representative species.

**Fig. 4:**
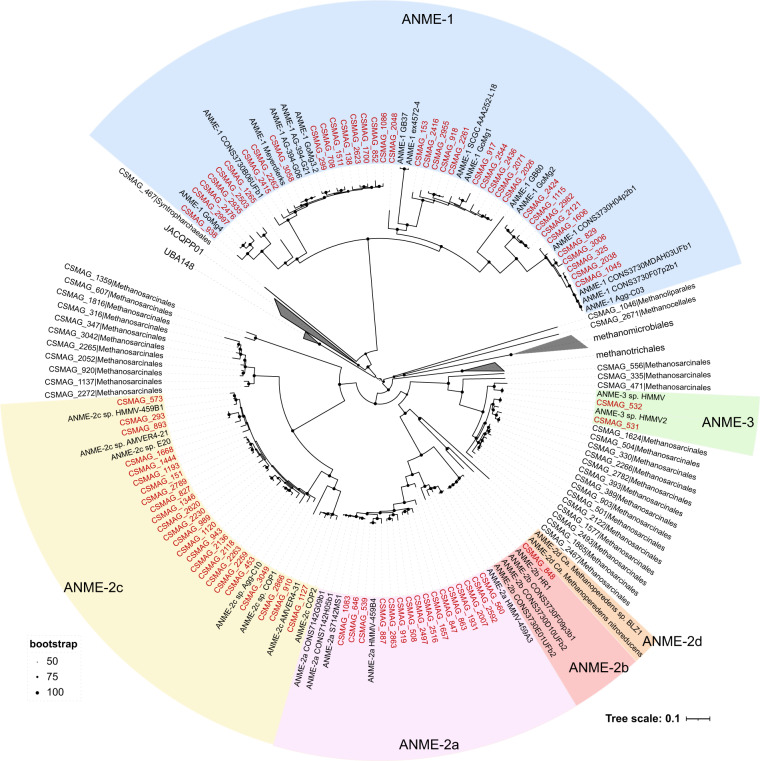
Phylogenetic tree of ANME genomes and related archaea. The phylogenetic tree was constructed from 41 previously published ANME genomes and 135 MAGs belonging to Halobacteriota from this study. The tree was constructed by the maximum likelihood method using a concatenated alignment of 53 conserved archaeal single-copy marker genes.

**Fig. 5:**
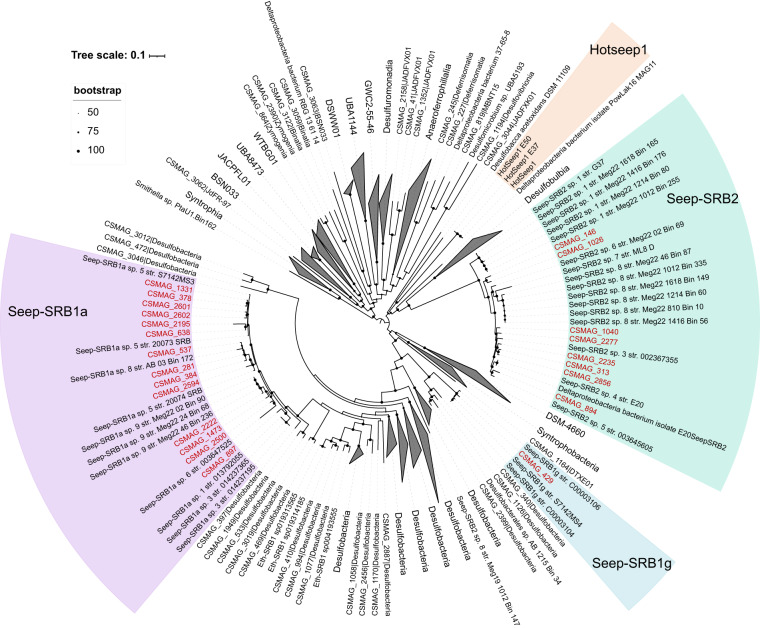
Phylogenetic tree of syntrophic SRB genomes. The tree was constructed by using 60 reference SRB genomes collected from previous studies and 327 MAGs assigned to Desulfobacterota from this study. The tree was constructed by the maximum likelihood method using a concatenated alignment of 120 conserved bacterial single-copy marker genes.

A total of 3,164 species-level MAGs were recovered in this study. The total mapping ratios of all these MAGs to clean reads of the 165 metagenomes averaged 27%. These MAGs covered various prokaryotic lineages spanning 113 phyla (97 bacterial and 16 archaeal). The phyla with the largest diversity of recovered species included Chloroflexota (n = 371), Proteobacteria (n = 335), Desulfobacterota (n = 306), Planctomycetota (n = 190), Patescibacteria (n = 152) and Bacteroidota (n = 151) and the archaeal phyla Halobacteriota (n = 129), Thermoplasmatota (n = 108), Thermoproteota (n = 98), Asgardarchaeota (n = 95) and Nanoarchaeota (n = 47) (Fig. 3b). Overall, ~94% of the recovered species are not represented in current databases (Fig. 3c), suggesting that cold seep sediments harbor a rich diversity of previously undescribed microbes. The non-redundant MAG catalog considerably expands the phylogenetic diversity and is an unparalleled genome resource of the cold seep microbiome. The compendium of ANME (Fig. 4) and syntrophic SRB MAGs (Fig. 5) expands the currently known diversity of these groups in cold seeps and will aid in expanding our understanding of the physiological basis of their interactions and their evolutionary histories.

## Methods

### Collection of metagenomes

Metagenomic datasets comprised 165 sediment samples (0 to 68.55 mbsf) collected from 16 globally distributed cold seep sites (Fig. 1a; Supplementary Table 1). These sites are as follows: Eastern North Pacific (ENP), Santa Monica Mounds (SMM), Western Gulf of Mexico (WGM), Eastern Gulf of Mexico (EGM), Northwestern Gulf of Mexico (NGM), Scotian Basin (SB), Haakon Mosby mud volcano (HM), Mediterranean Sea (MS), Laptev Sea (LS), Jiaolong cold seep (JL), Shenhu area (SH), Haiyang4 (HY4), Qiongdongnan Basin (QDN), Xisha Trough (XST), Haima seep (HM1, HM3, HM5, HM_SQ, S11, SY5, and SY6) and site F cold seep (RS, SF, FR, and SF_SQ). Paired-end sequencing data from ENP, SMM, WGM, NGM, HM, MS, LS and part of site F (RS and FR) were downloaded from the National Center for Biotechnology Information-Sequence Read Archive (NCBI-SRA) and European Bioinformatics Institute-European Nucleotide Archive (EBI-ENA) according to the accession numbers published in each study^8–10,22–26^. The remaining 106 metagenomic datasets used in this study were obtained from our previous publications^7,14,27–34^. Detailed sequencing information is available in Supplementary Table 1. These metagenomic samples were collected from a range of cold seeps, including oil and gas seeps, methane seeps, gas hydrates, asphalt volcanoes, and mud volcanoes. The samples were taken at various depths and under different redox conditions, from the oxic sediment-water interface to anoxic layers as deep as 68.55 meters below the sea floor.

### Contig assembly, gene prediction and gene catalog construction

Metagenomic sequence data were quality controlled using the Read_QC module (parameters: --skip-bmtagger) within the metaWRAP (v1.3.2) pipeline^35^ and fastp (v0.23.2; default parameters)^36^. After quality control, 9.5 Tb of clean reads remained for subsequent analyses. Clean reads from each cold seep sediment metagenome were assembled using MEGAHIT (v1.1.3 and v1.2.9, default parameters)^37^. In addition, co-assemblies were performed by combining metagenomes from all depths of each cold seep sediment using MEGAHIT (v1.1.3; parameters: --k-min 27 --kmin-1pass --presets meta-large)^37^. The assembly parameters are summarized in Supplementary Table 1. Contigs (length > 500 bp, n = 225,026,054) from individual assemblies and co-assemblies were used to predict protein-coding sequences (CDSs) with Prodigal (v2.6.3; parameter: -meta)^38^, which generated 373,051,862 protein sequences. These sequences were then clustered at 95% amino acid identity using CD-HIT (v4.8.1; parameters: -c 0.95 -aS 0.9 -g 1 -d 0)^39^. The cutoff of 95% amino acid identity was adopted to be consistent with the fact that members of the same microbial species generally share more than 95% average amino acid identity^40^. It should also be noted that the mixed-assembly approach used here, which combines data from single assemblies and co-assemblies, may enrich artificially long proteins to a certain extent^41^. This resulted in a non-redundant gene catalog comprising 147,289,169 representative clusters. The mapping-based mode of Salmon (v1.10.2)^42^ with a “meta-flag” was used to calculate the mapping rate of the non-redundant gene catalog in each metagenome.

### Functional annotation and taxonomic classification of the non-redundant gene catalog

The representative amino acid sequences from each cluster were functionally annotated using eggNOG-mapper (v2.1.9; default parameters)^43,44^. The functional annotations, including those for eggNOG 5.0, Pfam 33.1, KEGG, EC, GO, and CAZy, were derived from the eggNOG-mapper results. We found that 64% of the non-redundant genes had a hit in at least one of the following databases: eggNOG (n = 88,929,242; ~60%), Pfam (n = 85,404,569; ~58%), KEGG (n = 48,756,524; ~33%), EC (n = 27,619,712; ~19%), GO (n = 5,966,227; ~4%) and CAZy (n = 1,514,988; ~1%) (Fig. 2a,b). After analyzing the annotated genes based on the eggNOG database (Fig. 2c), the predominant category was “Function unknown” (n = 17,018,774). This category includes proteins that have not yet been characterized or for which there is insufficient information to assign a specific function. A total of ~40% of genes (n = 58,359,927; Fig. 2b) could not be assigned to an eggNOG orthologous group, similar to the percentage observed in the OM-RGC v2 (~39%)^21^ and higher than that in the GMGC v1 (~27%)^16^. According to the eggNOG database annotation, half of the genes (~51%), including 58,359,927 unannotated genes and 17,018,774 genes labeled as “Function unknown”, were functionally unidentified, suggesting that cold seeps harbor numerous unknown functional genes.

MMseqs2 taxonomy (v13.45111; parameter: --tax-lineage 1)^45^ was used to assign taxonomic labels to each representative amino acid sequence, using the GTDB R207 as a reference database^46^. The MMseqs2 taxonomy uses an approximate 2bLCA (lowest common ancestor, LCA) approach (--lca-mode: 2bLCA). A notable percentage of the non-redundant sequences (n = 44,441,531; ~30%) could not be classified as belonging to any prokaryotes in the GTDB, suggesting that these sequences may be attributed to novel prokaryotes (Fig. 2d). Approximately 9% (n = 13,154,825) of the non-redundant sequences could be identified only as either bacteria or archaea and could not be further classified at the phylum level (Fig. 2d). The results of taxonomic classification further confirm that this gene catalog contains many untapped genetic resources.

### Metagenomic binning and non-redundant MAG catalog construction

Assembled contigs were filtered by length (>1000 bp) for subsequent binning. BWA software (v0.7.17; BWA-MEM algorithm)^47^ was used to align short reads back to filtered contigs, with the alignment being sorted by SAMtools (v1.9)^48^. The contig depth profiles were produced using jgi_summarize_bam_contig_depths for running metabat2, maxbin2, SemiBin, Rosella and VAMB, while for running concoct, concoct_coverage_table.py was used. The binning process was performed using the metaWRAP binning module (v1.3.2; parameters: -metabat2, -maxbin2, -concoct, -universal)^35^, SemiBin with single_easy_bin mode (v1.4.0; default parameters)^49^, and Rosella (v0.4.1; default parameters; https://github.com/rhysnewell/rosella). The number of metagenomic samples collected from S11 (n = 13) and RS (n = 19) was larger than that obtained from other sites, making it computationally challenging to bin the co-assemblies of the samples from these sites. Thus, individual assemblies from the S11 and RS sites were concatenated and binned separately using the VAMB tool in “bin-split” mode (v3.0.2; parameters: --minfasta 200000 -o C)^50^. Afterward, the bins obtained with each binning tool were integrated and refined using the Bin_refinement module of the metaWRAP pipeline (v1.3.2; parameters: -c 50 -x 10)^35^. The completeness and contamination of refined bins were evaluated with CheckM (v1.2.1)^51^. Then, the resulting 8,654 MAGs were checked by GUNC (v1.0.5; default parameters)^52^ to remove genomes potentially containing chimerism based on “pass.GUNC”. All MAGs were dereplicated at the species level using dRep (v3.4.0; parameters: -comp 50 -con 10)^53^ with an average nucleotide identity (ANI) cutoff value of 95%. Representative genomes were selected based on the dRep scores derived from genome completeness, contamination and N50. A total of 3,164 MAGs with the highest dRep score from each species cluster were selected as the species representatives. MAGpurify software (v2.1.1; default parameters)^54^ was used to identify and remove putative contaminant contigs from each MAG based on the clade-markers, tetra-freq, gc-content, and clean-bin modules. Importantly, the resulting representative genomes should be considered population genomes within species^55^.

MAGs were taxonomically classified using the GTDB-Tk toolkit (v2.1.1)^56,57^ with default parameters against the R207 database. According to the taxonomic classification, four species clusters, with medium- or high-quality representatives (CSMAG_1499, CSMAG_2247, CSMAG_2329, and CSMAG_3128), were not assigned to any existing phylum. They did not cluster together and were included in different clades, exhibiting low relative evolutionary divergence values ranging from 0.32 to 0.43. These results suggest that these species belong to undescribed phyla. Additionally, 44 classes, 184 orders, 412 families, 1,043 genera and 2,984 species lacked classification assignments based on the GTDB R207 (Fig. 3c), representing potential novel lineages.

The coverage of each MAG was calculated using CoverM in genome mode (v0.6.1; https://github.com/wwood/CoverM; parameters: -min-read-percent-identity 0.95 -min-read-aligned-percent 0.75 -trim-min 0.10 -trim-max 0.90 -m relative_abundance) by mapping clean reads from the 165 metagenomes to all MAGs.

### Genomes for ANME and their syntrophic SRB

To explore the diversity of ANME lineages in global cold seep sediments, a phylogenetic tree was constructed that included 41 previously published ANME genomes^58–66^ and 135 MAGs belonging to Halobacteriota from this study. These published ANME genomes cover all of the currently described subclades: ANME-1, ANME-2a, ANME-2b, ANME-2c, ANME-2d, and ANME-3. To identify their syntrophic SRB, we constructed a phylogenetic tree of concatenated marker genes from 60 reference SRB genomes^6,23,67,68^ (including syntrophic SRB, namely, HotSeep-1, Seep-SRB2, Seep-SRB1a and Seep-SRB1g, and non-syntrophic SRB) and 327 MAGs assigned to Desulfobacterota from this study. The concatenated multiple sequence alignment of genomes based on 53 archaeal and 120 bacterial single-copy marker genes was produced via the identify and align workflow of GTDB-Tk (v2.1.0)^56^. The maximum likelihood tree was constructed using IQ-TREE (v2.2.0.3; parameters: -m MFP -B 1000)^69^. All produced trees were visualized using iTOL (v6)^70^.

A total of 81 ANME genomes were identified, namely, ANME-1 (n = 38), ANME-2a (n = 16), ANME-2b (n = 1), ANME-2c (n = 24), and ANME-3 (n = 2) (Fig. 4). In comparison, Chen *et al*.^15^ assessed the phylogenetic diversity of ANME MAGs from global methane seeps, which resulted in 47 species clustered into three subclades, including ANME-1a/b (n = 21), ANME-2a/b (n = 11), and ANME-2c (n = 15). The higher diversity of ANME captured here reflects the incorporation of all seep environments, not only those characterized by methane seepage. We also identified 23 syntrophic SRB MAGs (Fig. 5) spanning three clades (Seep-SRB2, n = 8; Seep-SRB1a, n = 14, and Seep-SRB1g, n = 1).

## Data Records

Details for the non-redundant gene catalog, the functional annotation and taxonomic classification for gene clusters, non-redundant MAGs, and phylogenetic trees are available in the Figshare repository^71^. All non-redundant MAGs are deposited in the NCBI database under BioProject PRJNA950938 (ref. ^72^) with the accession numbers detailed in Supplementary Table 2.

## Technical Validation

To maximize the number of genes and ensure the quality of the genes, we selected assembled contigs with a length greater than 500 bp to predict CDSs, as suggested in previous studies^17,73,74^. Then, we selected assembled contigs by length (>1000 bp) for metagenomic binning. The quality of MAGs was strictly controlled according to the following standards: (1) completeness >50% and contamination <10%; (2) genome sequences without potential chimerism (details in Supplementary Table 2); and (3) genome sequences without potential misassigned contigs.

## Usage Notes

The dataset compiled and analyzed in this study is the largest of its kind from cold seep sediment environments. Researchers could use the gene catalog of seeps to compare genes of interest to those in other habitats, such as glaciers, polar regions and hydrothermal vents, to study the habitat specificity of genes. The compendium of ANME could be used to investigate the distributional pattern of ANME archaeal communities in global cold seeps and ecological niche partitioning. Furthermore, the evolutionary and physiological basis of ANME-SRB interactions could also be explored.

### Supplementary information

Supplementary Table 1 and 2

## Data Availability

The present study did not use custom scripts to generate the dataset. The parameters and versions of all the bioinformatics tools used for the analysis are described in the Methods section. The code used to run each of the tools is available in the Figshare repository^71^.
